# BRCAA1 antibody- and Her2 antibody-conjugated amphiphilic polymer engineered CdSe/ZnS quantum dots for targeted imaging of gastric cancer

**DOI:** 10.1186/1556-276X-9-244

**Published:** 2014-05-19

**Authors:** Chao Li, Yang Ji, Can Wang, Shujing Liang, Fei Pan, Chunlei Zhang, Feng Chen, Hualin Fu, Kan Wang, Daxiang Cui

**Affiliations:** 1 Institute of Nano Biomedicine and Engineering, Key Laboratory for Thin Film and Microfabrication of Ministry of Education, Research Institute of Micro/Nano Science and Technology, Department of Instrument Science and Engineering, School of Electronics and Information, Shanghai Jiao Tong University, 800 Dongchuan Road, Shanghai 200240, People's Republic of China; 2 Department of Imaging Center, Affiliated Hospital of Xi'an Medical University, Xi'an 710077, People's Republic of China; 3 Department of Orthopedics, Xiangyan Hospital of Central South University, Changsha 410008, People's Republic of China

**Keywords:** CdSe/ZnS quantum dots (QDs), Amphiphilic polymer, BRCAA1 antibody, Her2 antibody, Cytotoxicity, Targeted imaging, Gastric cancer cell

## Abstract

Successful development of safe and highly effective nanoprobes for targeted imaging of *in vivo* early gastric cancer is a great challenge. Herein, we choose the CdSe/ZnS (core-shell) quantum dots (QDs) as prototypical materials, synthesized one kind of a new amphiphilic polymer including dentate-like alkyl chains and multiple carboxyl groups, and then used the prepared amphiphilic polymer to modify QDs. The resultant amphiphilic polymer engineered QDs (PQDs) were conjugated with BRCAA1 and Her2 monoclonal antibody, and prepared BRCAA1 antibody- and Her2 antibody-conjugated QDs were used for *in vitro* MGC803 cell labeling and *in vivo* targeted imaging of gastric cancer cells. Results showed that the PQDs exhibited good water solubility, strong photoluminescence (PL) intensity, and good biocompatibility. BRCAA1 antibody- and Her2 antibody-conjugated QD nanoprobes successfully realized targeted imaging of *in vivo* gastric cancer MGC803 cells. In conclusion, BRCAA1 antibody- and Her2 antibody-conjugated PQDs have great potential in applications such as single cell labeling and *in vivo* tracking, and targeted imaging and therapeutic effects' evaluation of *in vivo* early gastric cancer cells in the near future.

## Background

Gastric cancer is the second most common cancer and the third leading cause of cancer-related death in China [1,2]. It remains very difficult to cure effectively, primarily because most patients present with advanced diseases [3]. Therefore, how to recognize and track or kill early gastric cancer cells is a great challenge for early diagnosis and therapy of patients with gastric cancer.

We have tried to establish an early gastric cancer prewarning and diagnosis system since 2005 [4,5]. We hoped to find early gastric cancer cells *in vivo* by multimode targeting imaging and serum biomarker detection techniques [6-9]. Our previous studies showed that subcutaneous and *in situ* gastric cancer tissues with 5 mm in diameter could be recognized and treated by using multifunctional nanoprobes such as BRCAA1-conjugated fluorescent magnetic nanoparticles, Her2 antibody-conjugated RNase A-associated CdTe quantum dots, folic acid-conjugated upper conversion nanoparticles, RGD-conjugated gold nanorods, Ce6-conjugated carbon dots, and Ce6-conjugated Au nanoclusters (Au NCs) [10,11]. However, clinical translation of these prepared nanoprobes is always confounded by their *in vivo* biosafety. Development of safe and highly effective nanoprobes for targeted imaging and tracking of *in vivo* early gastric cancer cells has become our concern.

In the recent 10 years, quantum dots have been subjected to intensive investigations because of their unique photoluminescence properties and potential applications. So far, quantum dots have been used successfully in cellular imaging [12,13], immunoassays [14], DNA hybridization [15,16], and optical barcoding [17]. Quantum dots also have been used to study the interaction between protein molecules or to detect the dynamic course of signal transduction in live cells by fluorescence resonance energy transfer (FRET) [18,19]. These synthesized quantum dots have significant advantages over traditional fluorescent dyes, including better stability, stronger fluorescence intensity, and different colors, which are adjusted by controlling the size of the dots [20]. Therefore, quantum dots provide a new functional platform for bioanalytical sciences and molecular imaging. However, some studies also showed that some kinds of quantum dots exhibited toxic effects such as cytotoxicity, tissue toxicity, and *in vivo* residues [21,22]. How to develop safe quantum dots has become the concern of many scientists.

In our previous work, we also synthesized safe quantum dots such as Ag_2_S and AgSe [23,24] and used them for *in vitro* cell labeling and targeted imaging of *in vivo* gastric cancer cells. However, their fluorescence signals are too weak to be used for long-time imaging and single cell tracking [25]. How to prepare safe quantum dots with strong fluorescence signals has become a great challenge.

**Figure 1:**
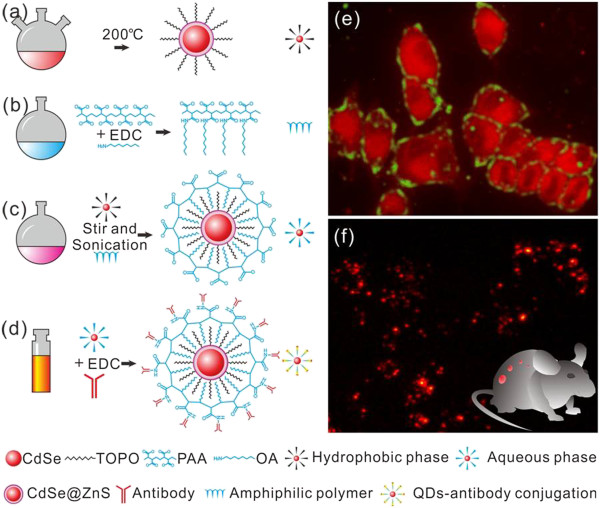
**Scheme of synthesis of CdSe/ZnS QDs, amphiphilic polymer, and coating process and their biological applications. (a)** Typical synthesis of CdSe/ZnS QDs in high temperature and cosolvent. **(b)** Synthesis of amphiphilic polymer: cross-linking PAA and OA by EDC. **(c)** Phase transfer of QDs from hydrophobic phase to hydrophilic phase by stirring and sonication. **(d)** Reaction scheme for coupling targeting antibody to PQDs by EDC. **(e)** Single molecule labeling and cell imaging with PQDs *in vitro*. **(f)** General labeled cancer cell with PQDs for imaging *in vitro* and *in vivo*.

In this study, as shown in Figure 1, we chose the CdSe/ZnS (core-shell) quantum dots (QDs) as prototypical materials, synthesized one kind of a new type of amphiphilic polymer including dentate-like alkyl chains and multiple carboxyl groups, and then used the prepared amphiphilic polymer to modify QDs. The resultant amphiphilic polymer engineered QDs (PQDs) were conjugated with BRCAA1 monoclonal antibody and Her2 antibody, and prepared BRCAA1 antibody- and Her2 antibody-conjugated QDs were used for *in vitro* labeling and *in vivo* targeted imaging of gastric cancer cells. Results showed that the amphiphilic PQDs exhibited good water solubility, strong photoluminescence (PL) intensity, and good biocompatibility. BRCAA1 antibody- and Her2 antibody-conjugated QD nanoprobes can specifically label gastric cancer MGC803 cells and realize targeted imaging of gastric cancer cells *in vivo* successfully.

## Methods

### Materials

Cadmium oxide (CdO, AR), stearic acid (98%), selenium powder, octylamine (OA, 99%), 1-hexadecylamine (HAD, 90%), and diethylzinc (ZnEt_2_) were obtained from Aladdin Co., Ltd. (Xi'an, China). Trioctylphosphine oxide (TOPO, 98%), trioctylphosphine (TOP, 95%), poly(acrylic acid) (PAA, molecular weight (MW) 1,800), 1-ethyl-3-[3-dimethylaminoporpyl] carbodiimide hydrochloride (EDC, 98.5%), and *N*-hydroxysuccinimide (NHS, 98%) were obtained from Sigma-Aldrich Co., Ltd. (St. Louis, MO, USA). Bovine serum albumin (BSA, 99.9%) was purchased from MP Biomedicals Company (Santa Ana, CA, USA). Bis(trimethylsilyl) sulfide ((TMS)_2_S) was purchased from Tokyo Chemical Industry Co., Ltd. (Tokyo, Japan). Liquid paraffin, chloroform, ethanol, hydrochloric acid (HCl), 2-(4-morpholino)ethanesulfonic acid (MES), *N*,*N*-dimethylformamide (DMF), paraformaldehyde, and Tween-20 were purchased from Sinopharm Chemical Regent Co., Ltd. (Shanghai, China).

### Synthesis of CdSe and CdSe/ZnS core-shell QDs

Highly luminescent core-shell CdSe/ZnS QDs were prepared in high temperature via the pyrolysis of organometallic reagents in a coordinating solvent [26-28]. We select 200°C with and without HAD for synthesis of green- and red-emitting CdSe QDs. The molar ratio of CdO/Se/stearic acid in liquid paraffin was 1:1:4, and the crude QD products were purified by chloroform and ethanol. For the ZnS shell, equal molar ratios of (TMS)_2_S and ZnEt_2_ as precursors of Zn and S, and TOP/TOPO were used, and 90°C was used for shell growth. The final core-shell product was repurified and redispersed into aliquot chloroform for later use. About 10 ml of deionized water was added to the solution to prevent evaporation of chloroform for long-period storage (see Additional file 1 for synthesis details of QDs).

### Synthesis and characterization of amphiphilic polymer

The amphiphilic polymer is synthesized as follows: in ambient temperature, 0.2 g of PAA (MW 1,800) was added to a flask containing 10 ml DMF. Under slight stirring for 1 h, 137 μl of OA was added, and the solution was continuously stirred for another 30 min. In an individual vial, 0.47 g EDC was dissolved in 0.5 ml DMF and injected to the reaction solution dropwisely. The reaction solution was mixed vigorously overnight to produce amphiphilic polymers (with 50% of the carboxylic acid functional groups modified with an aliphatic chain). Next, 0.25 M HCl was added drop by drop to the polymer solution under vigorous stirring, resulting in a milky and opaque colloid solution. The colloid was centrifuged at 8,000 rpm/min for 10 min. The supernatant was discarded, and the jelly-like precipitant was washed with 0.25 M HCl twice to remove any by-products and impurities. The final precipitate was collected and freeze dried to remove trace amounts of water, giving a dry, white powder. Fourier transform infrared (FTIR) spectroscopy (Equinox 55, Bruker, Karlsruhe, Germany) was used to verify the formation of amide bond and carboxylic groups.

### Preparation and characterization of amphiphilic polymers conjugated with QDs

An aliquot of amphiphilic polymer powder was resuspended in MES buffer (0.1 mol/l, pH 6.0) for later use. As-prepared QDs (200 μl, 0.15 mmol) dissolved in chloroform and amphiphilic polymer solution (2.0 ml, 0.45 mmol) were added to 8 ml of deionized water in an open container. The solution was stirred and sonicated for 30 min until the chloroform evaporated completely in the final products. Afterward, the hydrated colloid (polymer-coated QDs, PQDs) was further purified by size exclusion chromatography (Superdex 75, Pharmacia Biotech, AB, Uppsala, Sweden), yielding a transparent, homogeneous, and strong fluorescent solution.

After purification, the purified solution was then concentrated under reduced pressure using a rotary evaporator at approximately 15°C. For assessment of the size distribution and monodispersity of the PQDs, the primal QDs of CdSe, CdSe/ZnS, and purified PQDs were pipetted onto a carbon transmission electron microscopy (TEM) grid; the solvents were wicked away slowly after 15 min. For the PQDs, the grids were counterstained with a 1% phosphotungstic acid solution (pH adjusted to 6) for 30 s. The staining solution was wicked away similarly. All of the prepared grids were imaged (TEM, JEM-2100 F system, JEOL Ltd., Tokyo, Japan) and compared to determine size distribution of the QDs and the degree of polymer coating. For further size analysis, the as-prepared QDs and PQDs were measured using Zetasizer Nano ZSP (Malvern Instruments, Ltd., Worcestershire, UK). In addition, the optical properties of the prepared CdSe, CdSe/ZnS, and PQDs were measured using UV-visible and fluorescence spectrophotometer (Cary 50 Conc, Varian, Palo Alto, CA, USA; F-4600, Hitachi, Tokyo, Japan). The QD concentration was determined using Beer's law after measuring the absorbance value using spectrophotometry [29,30].

In order to estimate the surface charge and functional group character, we further characterized the polymer and PQDs by using 1% agarose gel electrophoresis. The agarose gel was prepared using standard techniques, and the prepared polymer and PQDs were added into the loading well. The gel was run in 0.5× TBE buffer (pH 8.0) for 30 min at 100 V and imaged with Tanon 2500 gel imaging system (Tanon, Shanghai, China) under 365-nm exciting light. Afterward, the gel was stained with lead acetate (1%) and potassium chromate (1%) for 5 min, respectively, and imaged in the Tanon 2500 system with white light to visualize the carboxyl group contained in the amphiphilic polymer. The migration rates of polymer and PQDs were compared to validate the success of QDs' surface coating.

### Effects of pH and ionic strength on the stability of PQDs

In order to evaluate the effects of a wide pH range and high salt concentration on the colloidal stability of the PQDs, the PQD colloids were dispersed in varied pH buffers, PQDs/buffer = 1:1 (*v*/*v*), and pH ranged from 2 to 13 (Additional file 1: details of preparation of a series of buffer solutions). The resulting PL spectra were background-corrected, integrated, and normalized to the intensity of PQDs in pH = 7, set as 100%. The stability effect of ionic strength was carried out as follows: dispersions of PQDs were placed in fluorescence cuvettes (1-cm optical path) containing an equal concentration of PQDs but various concentrations of sodium chloride. The lack of volumes was replenished with deionized water (pH = 7). The PL emission from PQDs without NaCl added was set to 100%. The resulting PL spectra were normalized to the emission form slat-free solution.

### Preparation of BRCAA1 antibody- and Her2 antibody-conjugated QD nanoprobes

The BRCAA1 monoclonal antibody was conjugated with red PQDs, whereas humanized Her2 monoclonal antibody was conjugated with green PQDs. The optimum mole ratio of PQDs to antibody is 5:3 [31]. The cross-linking reaction was done by using standard EDC-NHS procedure in ambient temperature and dark place for 2 h with continuous mixing. The mixture was then purified by chromatography (Superdex 75, Pharmacia Biotech, AB, Uppsala, Sweden) to remove the free antibody residues. The resultant BRCAA1 antibody- and Her2 antibody-conjugated PQDs were stored at 4°C for later use.

Afterward, the prepared PQDs and specific monoclonal antibody conjunction were analyzed in 8% sodium dodecyl sulfate-polyacrylamide gel electrophoresis (SDS-PAGE, Beyotime, Shanghai, China). The gel was run in a standard SDS buffer for 90 min at 120 V. Firstly, the gel was imaged with UV light to determine PQD position, and then, the gel was stained with Coomassie Brilliant Blue fast staining solution and imaged with white light to determine protein position.

The coupling rate of the PQDs and monoclonal antibody was estimated by a NanoDrop device (Thermo Scientific, Wilmington, DE, USA). Before coupling reaction, we measured the total concentration of monoclonal antibody. After coupling reaction, we estimated the monoclonal antibody concentration in the eluenting phase of chromatography and calculated the coupling rate according to the following equation:

$$\begin{array}{c} \text{Coupling}\;\text{rate}\;\left(\%\right)=(1-\text{Concentration}\;\text{of}\;\text{monoclonal}\;\text{antibody}\;\mathrm{in}\;\text{eluenting}\;\text{phase}/\text{Total}\;\text{concentration}\;\text{of}\;\text{monoclonal}\;\text{antibody})\\ \;\times 100. \end{array}$$

### BRCAA1 antibody- and Her2 antibody-conjugated QDs for targeted imaging of MGC803 cells *in vitro*

The overnight incubated MGC803 and GES-1 cells were fixed with 4% paraformaldehyde for 10 min and permeated with 0.5% (*v*/*v*) Tween-20 for 20 min. Then, these cells were blocked for 20 min in PBS containing 1% (*w*/*v*) BSA. After being washed with PBS twice, these cells were incubated at 4°C with BRCAA1 antibody- and Her2 antibody-conjugated QDs overnight. Unbound probes were removed by washing three times with PBS. Afterward, these cells were imaged under a fluorescence microscope (TS100, ×400, Nikon Co., Tokyo, Japan) and laser scanning confocal microscope in oil immersion objective (Nikon A1si+, ×1,000).

After attaining the fluorescence images, the gastric cancer cells were dissociated from the glass culture dish and sectioned as routine for TEM imaging.

### BRCAA1 antibody- and Her2 antibody-conjugated QDs for targeted imaging of gastric cancer cells *in vivo*

To quantitatively analyze the fluorescence intensity from PQD-labeled MGC803 cells, macro fluorescence images were acquired using PQD-labeled MGC803 cells which were diluted with PBS to a final concentration from 2 × 10^2^ to 2,048 × 10^2^ cells/200 μl. Afterward, 200 μl of the prepared cell solutions were added to polystyrene TC-treated 96-well microplates (Corning® Life Sciences, Corning, NY, USA, #3603). Fluorescence intensity was measured in a Bruker In-Vivo F PRO system (Bruker Corporation, UK), and the resulting background-corrected data was curve fitted to single exponentials. Signal curve fitting was done using the software Origin (OriginLab, Northampton, MA, USA; http://www.originlab.com/).

All of the following animal studies complied with current ethical considerations: Approval (SYXK-2007-0025) of the Institutional Animal Care and Use Committee of Shanghai JiaoTong University (Shanghai, China) was obtained. Nude mice (male, 18 to 22 g, 4 to 5 weeks old) were obtained from the Shanghai LAC Laboratory Animal Co. Ltd., Chinese Academy of Sciences (Shanghai, China, SCXK2007-0005), and housed in a SPF-grade animal center.

Pathogen-free athymic nude mice were housed in a vivarium accredited by our University. Male athymic nude mice (4 to 6 weeks old) were used to establish subcutaneous gastric cancer models; 1.5 × 10^6^ MGC803 cells suspended in 100 μl DMEM were subcutaneously injected into the left anterior flank area of each mouse. Four weeks later, tumors were allowed to grow to approximately 5 mm in diameter, and the prepared Her2 antibody-conjugated QDs (red, emission peak 657 nm) were injected into the mice via the tail vein for 6 h. Whole-animal imaging and *ex vivo* organ imaging were performed using the Bruker In-Vivo F PRO system. The excitation and emission filters were set to 410 and 700 nm (band pass, ±15 nm), respectively, and exposure time was set to 3 s. Collected images were analyzed using the imageJ software (NIH ImageJ; http://rsb.info.nih.gov/ij/), which uses spectral unmixing algorithms to separate autofluorescence from quantum dot signals.

## Results and discussion

### Characterization of synthesized CdSe, CdSe/ZnS QDs, and PQDs

**Figure 2:**
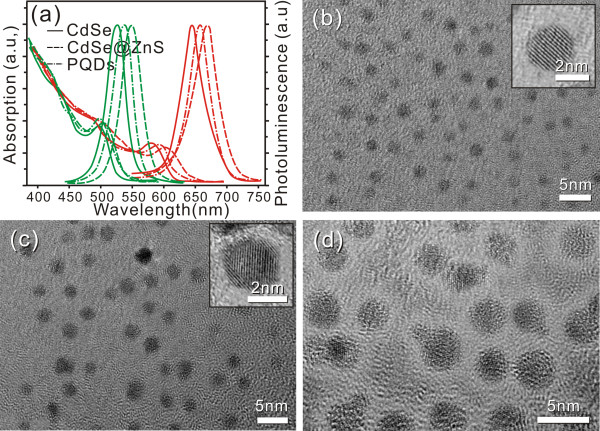
**Characteristics of synthesized CdSe, CdSe/ZnS, and amphiphilic polymer-coated QDs (PQDs). (a)** The absorbance and emission spectra of synthesized QDs. The green and red groups of lines represent absorbance (the curves from upper left to lower-middle part) and the photoluminescence (the curves with obvious protrusive shape) of the QDs at emission peaks of 526 and 644 nm, respectively. The solid line, dashed line, and dash-dot line indicate the core QDs, the core-shell structure QDs, and PQDs, respectively. **(b)** TEM image of CdSe cores. **(c)** TEM image of CdSe/ZnS core-shell prepared from CdSe. **(d)** TEM image of amphiphilic polymer-coated CdSe/ZnS core-shell QDs counterstained with 1% phosphotungstic acid solution. Insets in (b) and (c) showed the HRTEM images of the core and core-shell QD. (b,c,d represent red-colored QDs).

Different from our previous reports [3,32], the liquid paraffin and HDA were used as organic cosolvent to prepare the core CdSe QDs in this study. As shown in Figure 2a, synthesized QDs and PQDs exhibited strong fluorescence signal and narrow emission spectra. The emission peaks of synthesized CdSe, CdSe/ZnS, and PQDs are shown in Table 1. Figure 2b showed a typical TEM image of the CdSe core (reaction time 30 min, at 200°C). The QDs are observed to be spherically shaped, compact, and dense in structure, with a narrow diameter distribution of 4.3 nm approximately. The inset high-resolution transmission electron microscopy (HRTEM) image showed well-developed lattice fringes of the synthesized core structure. As shown in Figure 2c, the CdSe/ZnS QDs have a narrow size distribution of 4.8 nm in diameter. The existence of lattice planes on the HRTEM confirms the good crystallinity of the CdSe/ZnS core-shell structure. With the ZnS coating, the emission peak of CdSe/ZnS was shifted to a longer wavelength compared to that of the core, CdSe QDs (Table 1). The shell could not only enhance the core's anti-oxide ability, but also improved its stability and decreased the cytotoxicity [33-35]. The amphiphilic polymer-coated QDs were 5.4 nm in diameter (Figure 2d), while following 12- and 11-nm blueshift of the emission peak for red and green emission QDs, respectively (Figure 2a and Table 1). The green-colored QDs showed a similar TEM characterization with red emission color QDs (data not show).

**Table 1:** The emission peaks of synthesized CdSe, CdSe/ZnS, and PQDs (nm)

**Color**	**CdSe core**	**CdSe/ZnS core-shell**	**PQDs**
Green	526	549	538
Red	644	669	657

**Figure 3:**
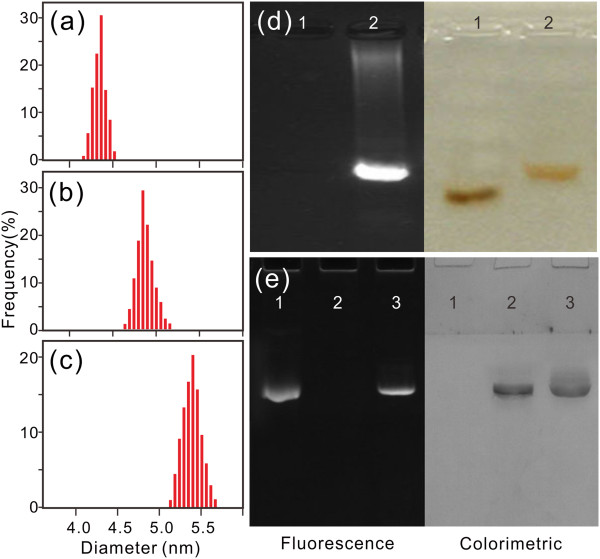
**Characteristics of synthesized QDs and PQDs (red).** The size histograms of synthesized **(a)** CdSe and **(b)** CdSe/ZnS in chloroform and **(c)** PQDs in MES buffer (pH = 6.0). **(d)** Electrophoretic images of synthesized amphiphilic polymer and PQDs. The left panel was taken under 365-nm UV lamp, and the right panel was taken in room light after staining with lead acetate and potassium chromate (lane 1, amphiphilic polymer; lane 2, PQDs). **(e)** SDS-PAGE results of PQDs (lane 1), antibody (BRCAA1, lane 2), and PQD-antibody conjugates (lane 3).

For biological application of QDs, the as-prepared core-shell QDs should be further coated with amphiphilic polymers or ligands that allow these nanomaterials to be transferred from the organic phase to water phase [36]. Different with PEG and other sulfhydryl compound- mediated aqueous solubility [37], in our experiments, we synthesized the amphiphilic polymer containing a dentate-like alkyl chain (hydrophobic) and the multiple carboxyl groups (hydrophilic) inlaid in the long aliphatic chains. The hydrophobic parts of the amphiphilic polymer could interact with the alkyl chain that coated the QD surface from TOPO; these two layers ‘bond’ to each other and form a hydrophobic protective structure that resists hydrolysis and enzymatic degradation even under complex *in vitro* or *in vivo* conditions [38]. The hydrophilic parts, in turn, are directed toward water and render the colloidal stability. Besides imparting aqueous solubility in a wide range of pH, the carboxyl groups can be used for further coupling chemistry with biological molecules or organic dyes such as carbodiimide (e.g., EDC)-based cross-linking and endowed it with potential applications of single molecule labeling, cellular imaging, or specific tissue mapping in clinical and biological practice [39].After a series of treatments were done as illustrated in Figure 1, we examined dispersibility of the prepared CdSe and CdSe/ZnS which were dissolved in chloroform and PQDs in MES buffer (pH = 6.0) using Zetasizer Nano ZSP. Figure 3a,b,c shows histograms of size distributions and aspect ratio from these synthesized samples (core emission peak 644 nm). This figure shows size distribution histograms of as-synthesized QD samples with an average size of (a) 4.3 ± 0.5 nm (CdSe in chloroform), (b) 4.8 ± 0.5 nm (CdSe/ZnS in chloroform), and (c) 5.4 ± 0.8 nm (PQDs in MES buffer).

The FTIR spectrum of the primary CdSe, CdSe/ZnS, and PQDs shows that (Additional file 1: Figure S1, details of FTIR) the peak of CdSe at 2,760 ~ 2,930 cm^-1^ is the characteristic symmetric and asymmetric methylene stretching (*v*C-H) that comes from the cosolvent material used in synthesis [40] (Additional file 1: Figure S1a). In the FTIR spectrum of CdSe/ZnS QDs (Additional file 1: Figure S1b), the peak at 1,183 cm^-1^ is the characteristic symmetric and asymmetric stretching vibrations from TOPO (*v*_P=O_) [37,41]. After transferring from the hydrophobic phase to the hydrophilic phase, for PQDs (Additional file 1: Figure S1c), many peaks emerged. The peak at 1,728 cm^-1^ is the vibration from C = O of the synthesized polymer (*v*C = O), and the peaks emerging at 1,609 and 1,310 cm^-1^ are the characteristic asymmetric and symmetric stretching vibrations from COO^-^ groups (*v*COO^-^) [42]. The difference in the FTIR spectrum of these QDs is an excellent evidence to prove that the PQDs had been successfully modified by the amphiphilic polymer.Figure 3d shows a comparison of the mobility shift of the amphiphilic polymer and 657-nm-emitting PQDs capped with the amphiphilic polymer. After 30 min of electrophoresis, the amphiphilic polymer cannot been seen in this UV condition (Figure 3d, left panel, lane 1). Meanwhile, the PQDs show a bright and narrow predominant band under the 365-nm UV light (Figure 3d, left panel, lane 2). This suggests that the synthesized PQDs are homogeneous. Afterward, the gel was stained with lead acetate and potassium chromate, and the carboxyl group was stained with lead chromate and had a dark yellow color. Under room light, the amphiphilic polymer and PQD (containing carboxyl groups) migrations can be seen clearly (Figure 3d, right panel).

### Stability of synthesized PQDs

**Figure 4:**
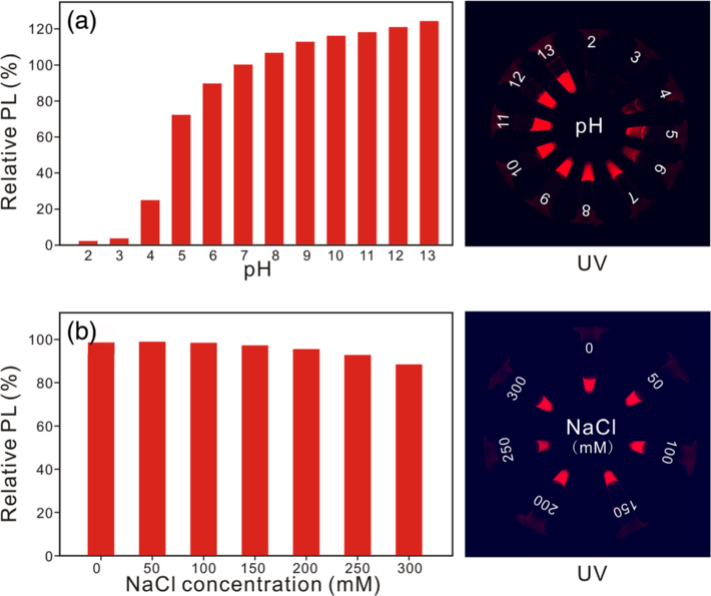
**Stability of synthesized PQDs in various pH values and different ionic strengths. (a)** Effect of pH on the photoluminescence of 623-nm-emitting PQDs. PQD colloids were dispersed in varied buffers, pH 2 ~ 13, PQDs/buffer = 1:1 (*v*/*v*). **(b)** Influence of increasing ionic strength on the photoluminescence of PQDs. The final sodium chloride concentrations varied from 0 to 300 mM (pH = 7.4).

In order to verify the long-term colloidal stability of the PQDs, we tested the PQD stability by a wide-range pH value. The images in Figure 4a show the relative photoluminescence intensity and fluorescence image of 657-nm-emitting PQDs in various pH values (the PL intensity in pH = 7 as the reference, 100%). We found that the strongly acidic condition (pH 4 or lower) rapidly led to a partial or complete fluorescence quenching of the PQDs, but no obvious agglomerate has been found. We surmise that this strongly acidic environment neutralized the surface negative charge of PQDs, resulting in agglomerate invisible to the naked eyes. The remaining PQDs were stable in weakly acidic to strongly basic pH conditions (pH 5 ~ 6 to approximately 13) without apparent fluorescence quenching for at least a 3-month period (Additional file 1: Figure S2, PL images of PQDs in different pH buffer with increasing span of time). We note that the pH stability of the present PQDs is comparable to that of QDs coated with DHLA or PMAA ligands [27,39,43] and is excellent, and our PQD preparation procedure possesses fewer steps and is more convenient for the synthesis of amphiphilic polymer and phase transfer.

In addition to the pH stability, we investigated the behavior of the PQDs in aqueous solutions with different ionic strengths. In the experiment, the PL properties of PQDs dispersed in PB buffer solutions at neutral pH were monitored, with NaCl concentration increased from 0 to 300 mM. Over the concentration range of NaCl, we observed little decrease in PL intensity and no change of the emission spectra for PQDs (Figure 4b, the PL intensity without NaCl added was set to 100%). This result is very similar with the previous reports [44,45]. These results of pH and ionic strength stability further highlight that the PQDs may be completely tolerant to intracellular and *in vivo* environments, where the ionic concentration is known to be less than 150 mM [46].

### The efficiency of PQDs conjugated with antibody

The prepared PQDs and PQD-antibody probe characteristics were assessed by 8% polyacrylamide gel electrophoresis (SDS-PAGE) [47]. In this experiment, the synthesized PQDs, monoclonal antibody, and PQD-antibody conjugation were added to specimen insertion ports, named lanes 1, 2, and 3, respectively. To avoid the acidic quenching effect on PQDs (the destaining solution contains acetic acid, based on the anterior results), after running with SDS buffer for 90 min, the gel was imaged on the Tanon 2500 gel imaging system with UV light (365 nm) in advance. To validate the coupling reaction, the gel was stained with Coomassie Brilliant Blue fast staining solution and washed with destaining solution. The stained gel was imaged again in white light. A comparison of the UV image with the image obtained by staining with Coomassie Blue is shown in Figure 3e. Apparently, in lane 1, the PQDs showed a clear bond which cannot be seen in bright fields (Figure 3e, left and right panels, lane 1). For monoclonal antibody, no signal can be detected in UV light but it is fairly visible in bright fields (Figure 3e, left and right panel, lane 2). However, in the conjugation of PQD-antibody, the band clearly can be seen both in UV light and bright fields; both of the migration ratios in different imaging conditions are identical (Figure 3e, left and right panels, lane 3). This result suggested that the conjugation between monoclonal antibody and PQDs is successful.

The mean coupling rates of BRCAA1 and Her2 were 75.52% and 73.37%, respectively, as shown in Table 2.

**Table 2:** Coupling rate measurements of PQD-antibody

	**BRCAA1**			**Her2**		
	**Total concentration (ng/ml)**	**The residue concentration (ng/ml)**	**Coupling rate (%)**	**Total concentration (ng/ml)**	**The residue concentration (ng/ml)**	**Coupling rate (%)**
1	10,000.0	2,204	77.96	10,000.0	2,582	74.18
2	10,000.0	2,749	72.51	10,000.0	2,865	71.35
3	10,000.0	2,566	74.34	10,000.0	2,773	72.27
4	10,000.0	2,177	78.23	10,000.0	2,309	76.91
5	10,000.0	2,545	74.55	10,000.0	2,785	72.15
Average			75.52			73.37

### Effects of PQDs on cellular viability

**Figure 5:**
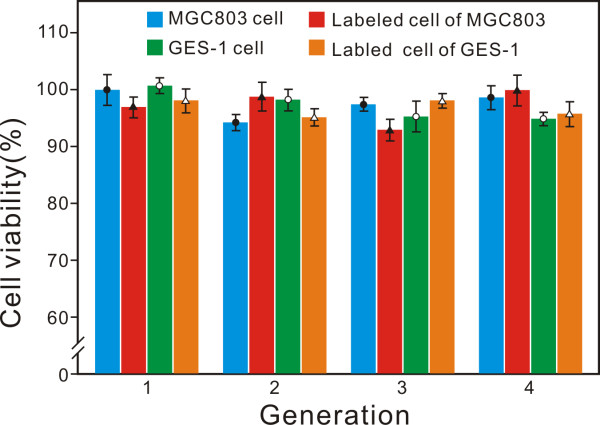
The MTT analysis results of MGC803 and GES-1 with and without PQD labeling.

In order to evaluate the influence of PQDs to living cells (MGC803 and GES-1), the labeled cells (non-specific labeling by endocytosis) were passaged parallel with the original cells (non-labeled). In each passage, the fissional and developmental abilities of these cells were estimated by MTT assay (repeated three times). Compared with the MTT results of PQD-labeled cells and the original cells, almost identical MTT values were gained in each generation (Figure 5). This consequence confirmed that the synthesized PQDs have negligible toxicity to the labeled cells and this is the essential requirement for further clinical applications [48,49].

### BRCAA1 monoclonal antibody-conjugated QDs for *in vitro* targeted imaging

**Figure 6:**
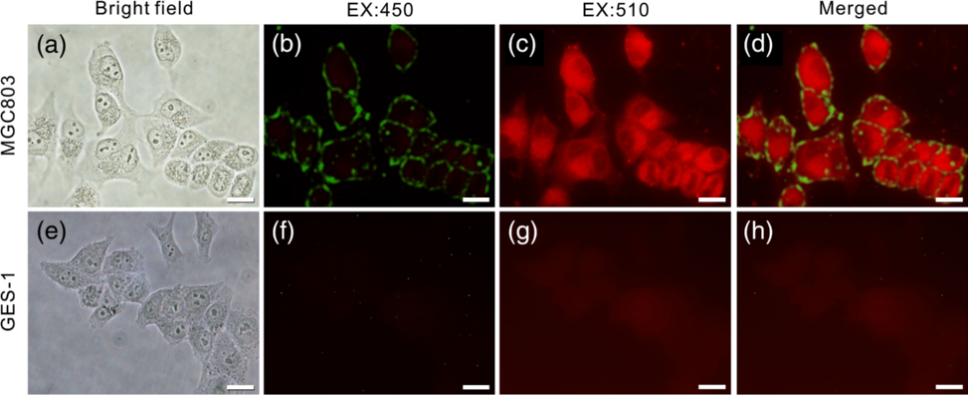
**PQD-antibody probes for targeted imaging of *in vitro *MGC803 cells. (a-d)** Bright-field and fluorescence images of gastric cancer MGC803 cell line; the cells were incubated at 4°C with PQD-antibody probes (BRCAA1 and Her2) in 1% BSA overnight (similarly hereinafter), excited with 450 and 510 nm for Her2 and BRCAA1 probes, respectively, and exposure time was 15 s. **(e-h)** Bright-field and fluorescence images of human fetal gastric epithelial GES-1 cell line; fluorescence exposure time was 60 s. Scale bars are 25 μm.

BRCAA1 antigen is a specific protein for the intracellular epitope of histone deacetylase complex subunit SAP180 expressed in the cytoplasm of the breast cancer cell line MCF-7 and gastric cancer cell line MGC803 [3]. Moreover, several research results confirmed the overexpression of Her2 in gastric cancer and some other aggressive disease [50]. Therefore, we choose these two monoclonal antibodies (BRCAA1 conjugate to red PQDs and Her2 conjugate to green PQDs) as single molecular probes to image gastric cancer cells. In addition, because both expressing (MGC803 cell) and non-expressing (GES-1 cell) cells can be simultaneously visualized in a given microscopic field of view, the non-expressing cells could serve as a good control [51].The targeted imaging results are shown in Figure 6. Each bright-field image shows multiple cells (Figure 6a,e), but only MGC803 cells expressing specific protein (antigen) of BRCAA1 and Her2 were labeled with PQD-anti-BRCAA1 (red) and PQD-anti-Her2 (green) probes and presented evenly fluorescent signal in the cytoplasm (BRCAA1) and membrane (Her2) (Figure 6b,c,d). In the GES-1 cell without expression of BRCAA1 and Her2 antigens, very weak or no apparent signals were detected (Figure 6f,g,h). This result indicated that the synthesized PQD-antibody probes are relatively specific for the established targets. This correlation demonstrates that the single molecule expressed in the intracellular environment or membrane can be targeted and imaged by PQD-antibody probes. This approach can thus be extended to specifically label target proteins or cell types to visualize their interactions in fixed cells and pathological sections.

**Figure 7:**
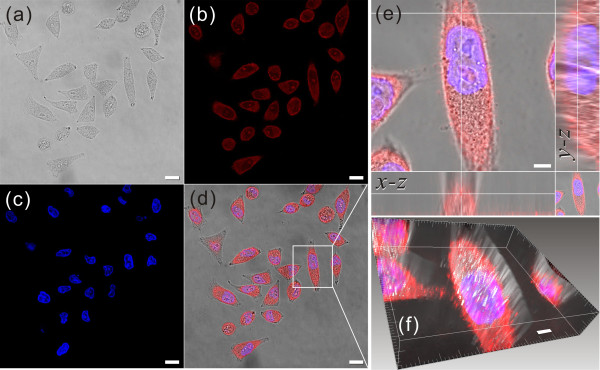
**Confocal micrographs of MGC803 cell target-labeled with the BRCAA1-antibody PQD probes. (a)** Bright field, **(b)** cytoplasm labeled by PQDs, **(c)** nucleus stained by DAPI, **(d)** cosituated picture of cells and fluorescence. **(a-d)** Scale bars are 25 μm. **(e)***Z*/*X*- and *Z*/*Y*-sections reconstructed from a confocal series through representative cells. **(f)** Three-dimensional reconstruction of representative cells. **(e-f)** Scale bar represents 5 μm. Fourteen sections of 990 nm were taken for each series, and *Z*-sections were reconstructed with Imaris™ software. *Z*-sections were taken at a line running through the midpoint of the *XY* plane.

**Figure 8:**
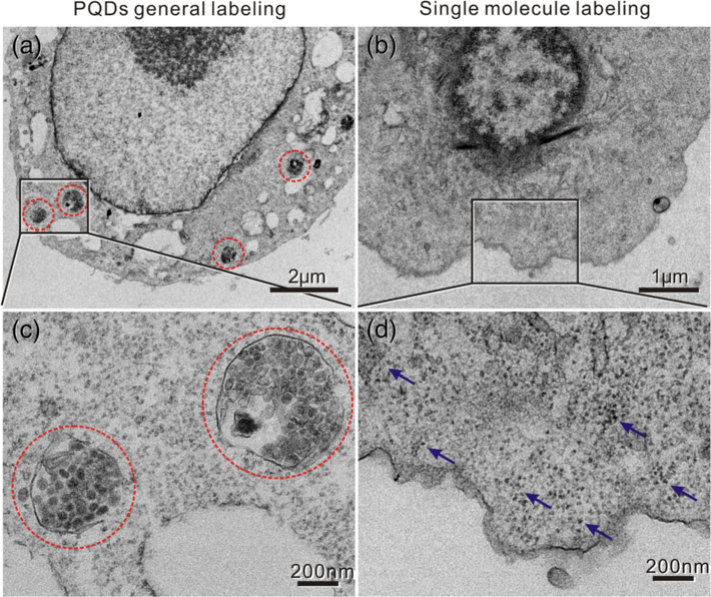
**TEM images of endocytosis of PQDs and single molecule labeling with PQD-antibody probes in MGC803 cell. (a, c)** TEM images of general labeling with PQDs; the red circles enclose PQD granules endocytosed by MGC803 cells. **(b, d)** Targeted single molecule labeling with synthesized PQD-antibody probes; the blue arrows pointed out the evenly distributed biomolecule probes in the cytoplasm of the MGC803 cell.

To confirm the application of the prepared PQD-antibody probe for gastric cancer cell imaging, the gastric cancer MGC803 cell was labeled with the PQD-anti-BRCAA1 probe as mentioned above. Then, the cell was observed by confocal laser microscopy. Figure 7 shows that the cytoplasma was evenly labeled by the PQD-anti-BRCAA1 probe to red (Figure 7b) and the cell nuclei were stained by DAPI to blue (Figure 7c). By means of *Z*/*X*- and *Z*/*Y*-sections constructed from the confocal series, it can be seen that the synthesized PQDs were homogeneously distributed in the cell cytoplasma (Figure 7e). Furthermore, the three-dimensional reconstruction of representative cells showed that the PQDs were predominantly distributed in the cytoplasm and not the nucleus because the BRCAA1 protein was expressed mainly in the cytoplasm (Figure 7f). These results indicated that the synthesized PQD-anti-BRCAA1 probe could penetrate the cellular membrane and bind with the protein molecule expressed in the cytoplasm of the MGC803 cell. Additionally, these fluorescence images also further elucidated that the synthesized PQD-antibody probes could target label the single molecule expressed in the gastric cancer MGC803 cell line efficiently.We also examined the endocytosis of PQDs and prepared nanoprobes such as BRCAA1 antibody-PQDs in MGC803 cells. In endocytosis, the PQDs were distributed in the cytoplasm as granules and colocalized almost completely in endocytic vesicles (red circles in Figure 8a,c); this indicates that the PQDs were internalized by endocytosis pathway. Regarding targeted labeling, the BRCAA1 antibody-PQD probes were distributed evenly in the cytoplasm (blue arrows in Figure 8b,d), and this was consistent with microscopic and confocal images mentioned above. The TEM images certified that the synthesized PQD-antibody probes can target and image the MGC803 cell specially.

### BRCAA1 monoclonal antibody-conjugated QDs for *in vivo* targeted imaging

**Figure 9:**
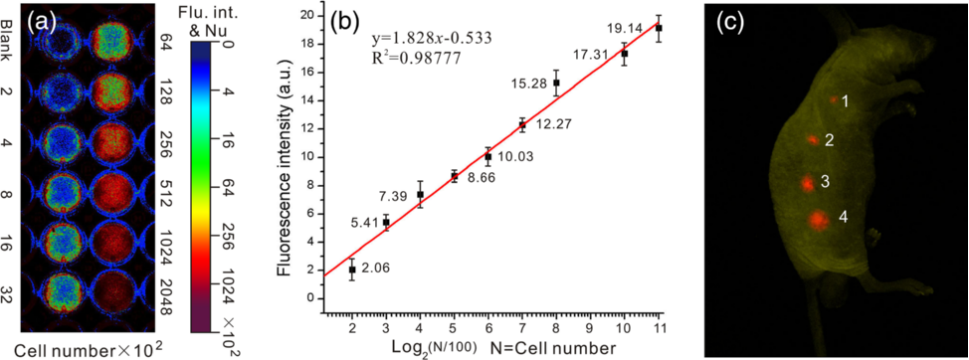
**Sensitivity and capability of PQDs (red)-labeled MGC803 cell imaging in live animals. (a, b)** The quantitative analysis of fluorescence of PQD-labeled MGC803 cells showed a linear relationship (*R*^2^ = 0.98777) between fluorescence intensity and cell numbers. **(c)** Fluorescence imaging of different amounts of PQD-labeled MGC803 cells injected subcutaneously in a mouse (cell numbers of 32× 10^2^, 128× 10^2^, 512× 10^2^, and 2,048 × 10^2^ corresponded to the sites 1, 2, 3, and 4 marked in the image; excitation filter 410 nm, emission filter 700 ± 15 nm, band pass).

For *in vivo* imaging, it is important to estimate the parameters of fluorescence intensity and the labeled cells; after that, the optimum number of the labeled cells can be decided for *in vivo* imaging. From Figure 9a,b, we can see that there is a linear increase with the number of PQD (red)-labeled MGC803 cells from 2 × 10^2^ up to 2,048 × 10^2^, but the system appears to become saturated when greater numbers of cells are introduced.

As shown in Figure 9c, after subcutaneous injection of different amounts of PQD-labeled MGC803 cells in the athymic nude mouse, the fluorescence signals were observed in the injection sites and there was a steady increase of the fluorescence intensity in the injection sites with the increment of PQD-labeled MGC803 cells. As can be seen in injection site 1, merely 32 × 10^2^ PQD-labeled cells could provide a significant fluorescence signal. The fluorescence signal of *in vivo* imaging shows that MGC803 cells were successfully labeled with PQDs.

**Figure 10:**
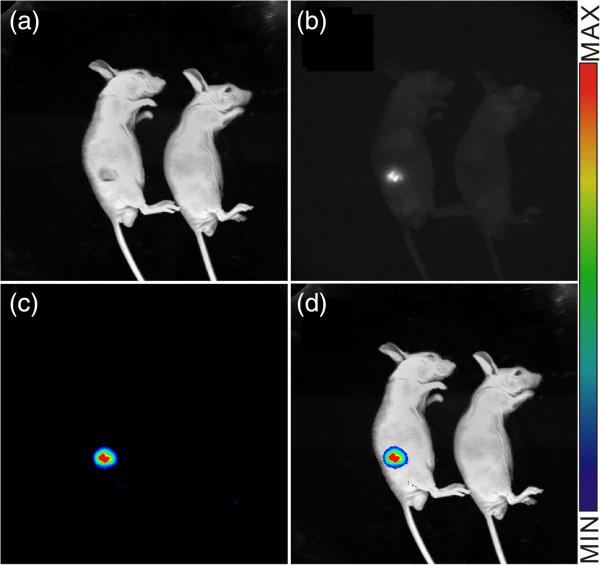
**Targeted imaging of gastric cancer in nude mice model by BRCAA1 monoclonal antibody-conjugated QDs. (a)** Nude mouse model loaded with MGC803 cells and control mouse. **(b)** Targeted imaging of *in vivo* gastric cancer under dark visual field. **(c)** The fluorescence signal of *in vivo* gastric cancer (pseudocolor). **(d)** Colocalization image of bright field and fluorescence signal.

After BRCAA1-antibody-conjugated PQD nanoprobes were injected into nude mice via the tail vein for 24 h, as shown in Figure 10, most of the prepared QD nanoprobes accumulated in the tumor site. This result showed that the synthesized nanoprobes can be successfully used for targeted imaging of *in vivo* gastric cancer in gastric cancer-bearing nude mice models.

## Conclusion

In conclusion, BRCAA1 monoclonal antibody- and Her2 antibody-conjugated amphiphilic polymer-modified core-shell CdSe/ZnS quantum dots were successfully prepared, exhibited good biocompatibility and strong stable fluorescence signals, and were successfully used for *in vitro* and *in vivo* targeted imaging of gastric cancer MGC803 cells. High-performance BRCAA1 antibody- and Her2 antibody-conjugated amphiphilic polymer-modified core-shell CdSe/ZnS quantum dot nanoprobes exhibit great potential in applications such as molecular imaging and therapeutic effect evaluation of early gastric cancer in the near future.

## Competing interests

The authors declare that they have no competing interests.

## Authors’ contributions

CDX carried out the experimental design and revised the manuscript. LC and YJ carried out the synthesis, analysis of QDs and amphiphilic polymer, and cell imaging and drafted the manuscript. WC and LSJ carried out the antibody coupling and cell culture. ZCL and CF participated in the synthesis and analysis of QDs. PF, WK, and FHL conceived the cell labeling process. All authors read and approved the final manuscript.

## Supplementary Material

Additional file 1 **Supplementary data.** A file showing data on the preparation of CdSe and CdSe/ZnS quantum dots and preparation for a series of buffer solutions, and images of FTIR spectrum of synthesized CdSe, CdSe/ZnS, and PQDs and PL spectra for a set of PQDs capped with the amphiphilic polymer in different buffers at pH 5~13. Click here for file
