# Supplementary material for: BRCAA1 antibody- and Her2 antibody-conjugated amphiphilic polymer engineered CdSe/ZnS quantum dots for targeted imaging of gastric cancer
Source: Nanoscale Res Lett. 2014 May 19;9(1):244. doi: 10.1186/1556-276X-9-244 (PMC4043660; doi:10.1186/1556-276X-9-244)
Supplement: Additional file 1 — Supplementary data. A file showing data on the preparation of CdSe and CdSe/ZnS quantum dots and preparation for a series of buffer solutions, and images of FTIR spectrum of synthesized CdSe, CdSe/ZnS, and PQDs and PL spectra for a set of PQDs capped with the amphiphilic polymer in different buffers at pH 5~13. [file 1556-276X-9-244-S1.doc]

**Additional file**

**BRCAA1- antibody and Her2 antibody -conjugated amphiphilic polymer engineered CdSe/ZnS quantum dots for targeted imaging of in vivo gastric cancer**

Chao Li, Yang Ji, Can Wang, Shujing Liang, Fei Pan, Chunlei Zhang, Feng Chen, Hualin Fu, Kan Wang, Daxiang Cui

**S1. Preparation of CdSe and CdSe/ZnS quantum dots**

Briefly, with the nitrogen protection, 79 mg selenium powder was dissolved in 50 ml liquid paraffin in a three neck flask and the temperature was increased to 200℃ gradually. After vigorous stirring for 1 hour, the mixture became bright and clear, let the mixture cool to approximately 80℃ with continue stirring. In another flask, 1.28 g CdO and 11.4 g stearic acid were dissolved in 10ml liquid paraffin at 160℃ with stirring and protection of nitrogen. For green emission color QDs, extra 0.5g HAD was added to prepare Cd precursor solution. When the mixture turned to bright yellow, rapidly inject the cooled solution of Se precursors into the hot flask that containing Cd precursors and quickly increase the mixture temperature to 200℃ for 90 minutes. For green emission color QDs, the reaction time limited to 5min. After that, the mixture temperature was decreased to 60℃, equal volume chloroform was added into it and mixed thoroughly. The mixture was centrifuged at 8000rpm/min for 10 min; the unreacted material and coordinating solvent (liquid paraffin and stearic acid) were discarded in the superstratum (solid or semisolid). Equal volume of ethanol was added to the collected underlayer and mixed evenly. After another centrifuge, the precipitation was washed extra time with ethanol. The precipitation was dried to brown powder in a hot air circulating oven and stored in dark environment.

In order to improve the optical properties of QDs, an additional semiconductor shell (zinc sulfide, ZnS) should be coated on CdSe nanocrystals. In this experiment, ZnEt_2_ and (TMS)_2_S were used as the Zn and S precursors. The amounts of Zn and S precursors needed to grow a ZnS shell for desired thickness were calculated as reference. In short, under an atmosphere of nitrogen, a reaction flask containing 2.5 g TOPO and 1.25g HAD been heated to 135℃ for 1 hour with vigorous stirring. In a 50ml centrifugal tube, 2g CdSe QDs was dispersed in 20ml chloroform and sonicated for 1 hour. After that, the QDs solution was injected to the reaction flask and maintained the temperature in 135℃ for 3 hours to evaporate chloroform completely. In a separate vial, 90ul (TMS)_2_S and 82ul of ZnEt_2_ were added into 2.5ml TOP and mixed evenly in a inert atmosphere glove box. Then, the precursor mixture was loaded from the vial into a syringe and injected slowly to the reaction flask when the temperature reached to 180℃ (at a rate of about 0.2 ml/min). An inorganic passivating shell of ZnS was then grown on the nanocrystals using organometallic precursors at ~180℃, as described in literature. Once the addition was complete, lower the solution temperature to 90 ℃ and annealing the mixture under stirring for several hours. After that, a 5 ml aliquot of chloroform was added to the mixture to prevent the TOPO from solidifying upon cooling to room temperature. Then, the reaction mixture was redissolved in 10 ml of chloroform and transferred to 50ml centrifugetube. Afterward, equal volume ethanol was added to the mixture and centrifuged in 5000rpm/min for 10min. The supernatant was discarded, and precipitate was washed with ethanol twice to remove non-reacted precursor and impurities. The final product was baked and quantified. The final core-shell production was redispersed into aliquot chloroform. About 10ml deionized water was added to the solution to seal evaporation of chloroform for long period store.

**S2. P**reparation for a series of buffer solutions (Total 20ml for each)

| pH | 0.2M glycine (ml) | 0.2M HCl (ml) | Deionized water (ml) |
| --- | --- | --- | --- |
| 2* | 5 | 4.4 | 10.6 |
| 3* | 5 | 1.14 | 13.86 |
|  | *Glycine HCl buffer |  |  |
|  | 0.2M Na_2_HPO_4_ (ml) | 0.1M Citrate (ml) |  |
| 4^△^ | 7.71 | 12.29 | / |
| 5^△^ | 10.3 | 9.7 | / |
| 6^△^ | 12.63 | 7.37 | / |
| 7^△^ | 16.47 | 3.53 | / |
| 8^△^ | 19.15 | 0.85 | / |
|  | ^△^Phosphate Citrate buffer |  |  |
|  | 0.2M glycine | 0.2M NaOH |  |
| 9^▲^ | 5 | 0.88 | 14.12 |
| 10^▲^ | 5 | 3.2 | 11.8 |
|  | ^▲^Glycine NaOH buffer |  |  |
|  | 0.1M Na_2_CO_3_(ml) | 0.1M NaHCO_3_(ml) |  |
| 11^★^ | 19 | 1 | / |
|  | ^★^Crabonate Bicarbonate buffer |  |  |
|  | 0.05M Na_2_HPO_4_ (ml) | 0.1M NaOH (ml) |  |
| 12^☆^ | 10 | 5.38 | 4.62 |
|  | ^☆^Phosphate NaOH buffer |  |  |
|  | 0.2 M KCl | 0.2M NaOH |  |
| 13^●^ | 5 | 13.2 | 1.8 |
|  | ^●^KCl NaOH buffer |  |  |


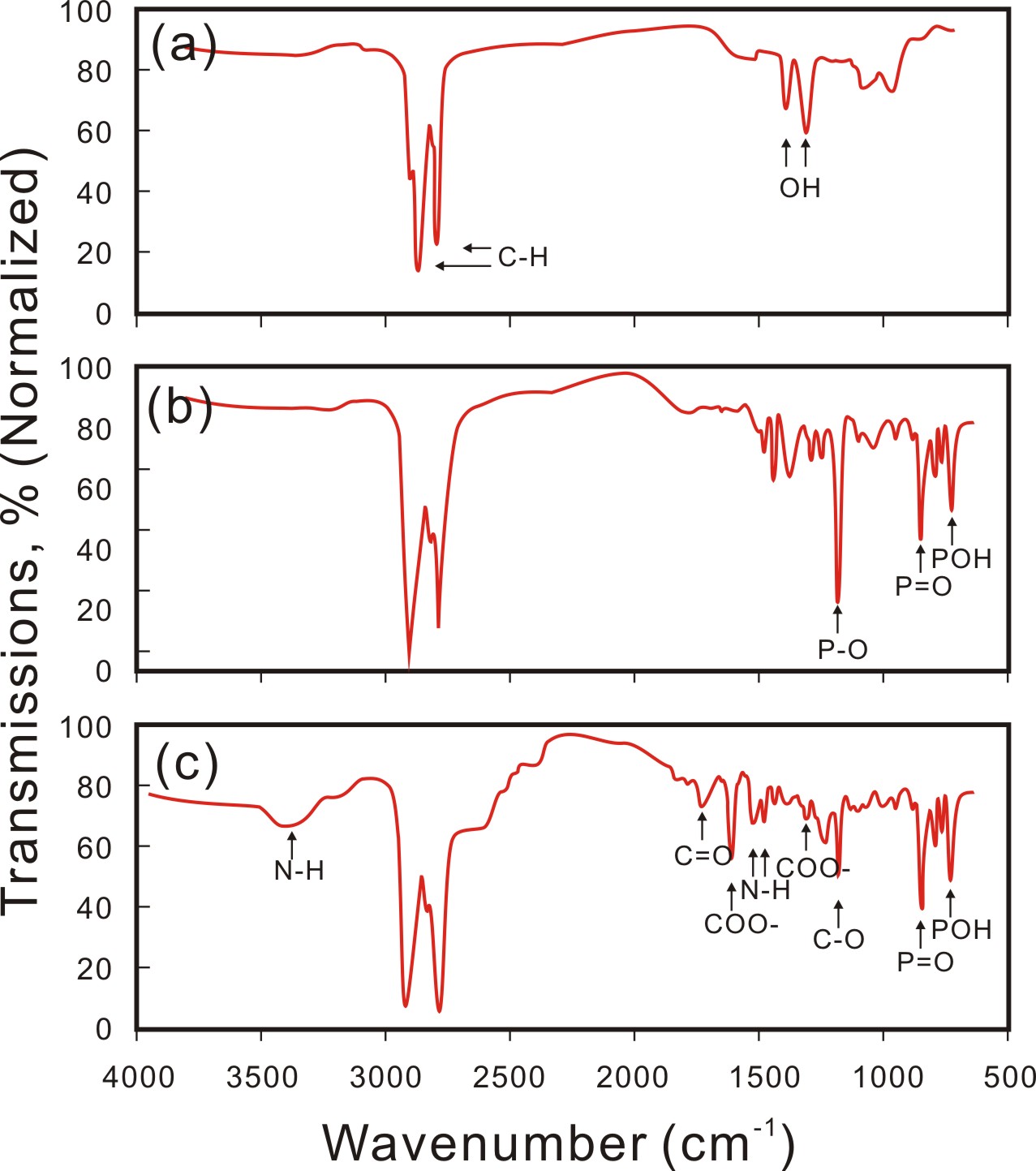


**Figure S1.** FTIR spectrum of synthesized CdSe (A), CdSe/ZnS (B) and PQDs (C).

Figure S1A shows the FTIR spectrum of the primary CdSe QDs. The peak at 2760~2930 cm^-1^ is the characteristic symmetric and asymmetric methylene stretching (*v*C-H). These peaks come from the multiple alkyl compounds, paraffin, the cosolvent material used in synthesis. Another two tiny peaks at 1309 and 1378cm^-1^ is the characteristic asymmetric methylene stretching, and these peaks may come from high temperature oxidation in fabrication procedure. In figure 3E, the FTIR spectrum of CdSe/ZnS QDs, the peak in 1183cm^-1^ is the characteristic symmetric and asymmetric stretching vibrations from TOPO( *v*_P=O_). But the peaks distributed in 721 and 853cm-1 are the weak P=O and POH stretching after TOPO treatment ( *v*_P=O_ and *v*_P-O_). After surface modification, for the PQDs (Fig. 3F), many peaks emerged. The distinctive peaks in 3417cm^-1^ and 3244cm^-1^ are the asymmetric (3417) and symmetric (3244) N-H stretching peaks that merged together and give this broad peak at 3100~3500cm^-1^. The peak in 1728cm^-1^ is the vibration from C=O of synthesized polymer (*v*C=O) and the peaks emerged in 1609cm^-1^ and 1310cm^-1^ are the characteristic asymmetric and symmetric stretching vibrations from COO^-^ groups (*v*COO^-^). The following peaks arose in 1476 and 1523cm^-1^ are the characteristic peaks of N-H group bending vibration, symmetric and asymmetric ring breathing. Meanwhile, the peak appeared in 1179cm^-1^ is the symmetric vibration from C-O group of synthesized polymer ( *v*C-O). Conclusively, the difference in FTIR spectrum of these QDs is an excellent evidence to prove the PQDs had been successfully modified by the amphiphilic polymer.


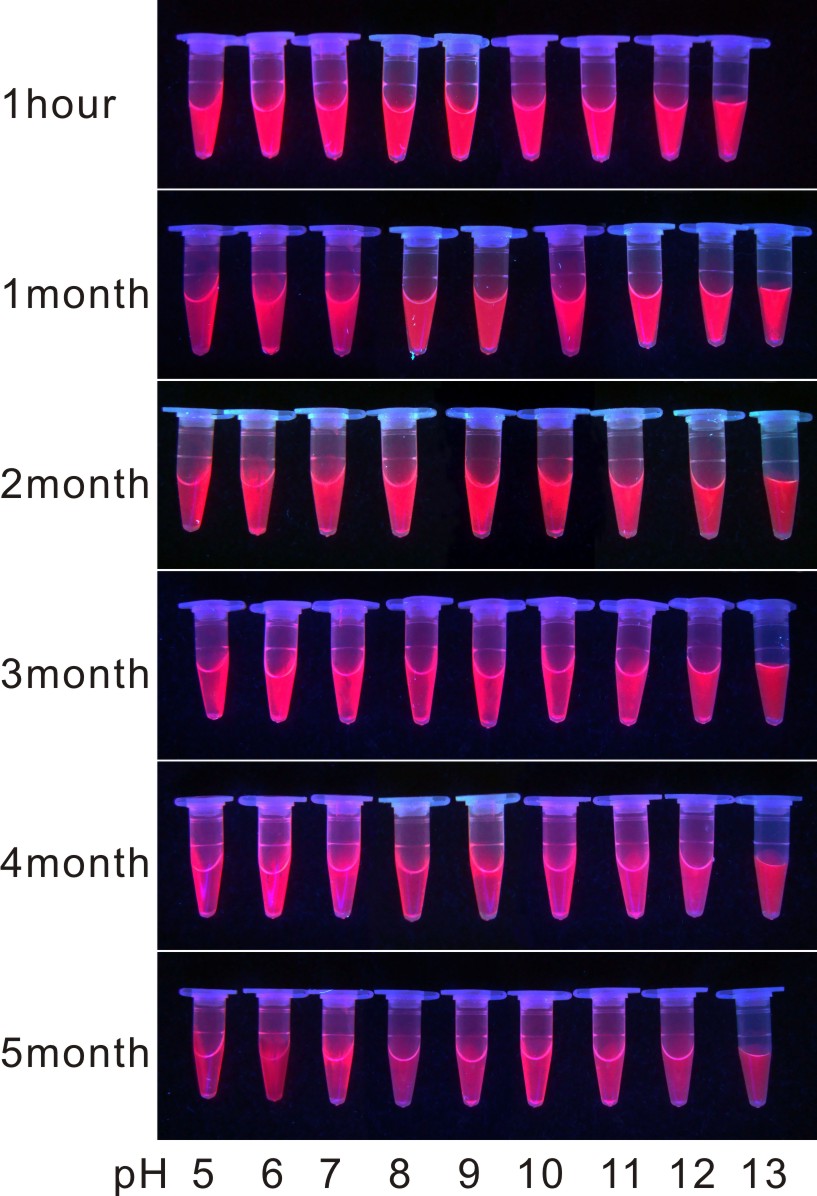


**Figure S2.** PL spectra for a set of PQDs capped with the amphiphilic polymer in different buffers at pH 5~13 (1:1, vol/vol). The 623 nm emitting PQDs were used and excited with a UV lamp at 365 nm. Images were taken in 1hour and ∼5 months after sample preparation.

**Reference**

1. Watson A, Wu X and Bruchez M, **Lighting up cells with quantum dots**, *Biotechniques*, 2003, **34**, 296.

2. Dabbousi B, Rodriguez-Viejo J, Mikulec F V, Heine J, Mattoussi H, Ober R, Jensen K and Bawendi M, **(CdSe) ZnS core-shell quantum dots: synthesis and characterization of a size series of highly luminescent nanocrystallites**, *J Phys Chem B*, 1997, **101**, 9463-9475.

3. Yu W W, Chang E, Falkner J C, Zhang J, Al-Somali A M, Sayes C M, Johns J, Drezek R and Colvin V L, **Forming biocompatible and nonaggregated nanocrystals in water using amphiphilic polymers**, *J Am Chem Soc*, 2007, **129**, 2871-2879.

4. Deng Z, Cao L, Tang F and Zou B, **A new route to zinc-blende CdSe nanocrystals: mechanism and synthesis**, *J Phys Chem B*, 2005, **109**, 16671-16675.

5. Mei B C, Susumu K, Medintz I L, Delehanty J B, Mountziaris T and Mattoussi H, **Modular poly (ethylene glycol) ligands for biocompatible semiconductor and gold nanocrystals with extended pH and ionic stability**, *J Mater Chem*, 2008, **18**, 4949-4958.

6. Permadi A, Fahmi M Z, Chen J-K, Chang J-Y, Cheng C-Y, Wang G-Q and Ou K-L, **Preparation of poly (ethylene glycol) methacrylate coated CuInS2/ZnS quantum dots and their use in cell staining**, *RSC Adv*, 2012, **2**, 6018-6022.

7. Bernier M-H, Levy G J, Fine P and Borisover M, **Organic matter composition in soils irrigated with treated wastewater: FT-IR spectroscopic analysis of bulk soil samples**, *Geoderma*, 2013, **209**, 233-240.
